# Crystal structure of aqua­(citric acid)(hydrogen citrato)calcium monohydrate, [Ca(HC_6_H_5_O_7_)(H_3_C_6_H_5_O_7_)(H_2_O)]·H_2_O, from synchrotron X-ray powder data, and DFT-optimized crystal structure of existing calcium hydrogen citrate trihydrate, [Ca(HC_6_H_5_O_7_)(H_2_O)_3_]

**DOI:** 10.1107/S2056989020012864

**Published:** 2020-09-25

**Authors:** James A. Kaduk

**Affiliations:** a Department of Physics, North Central College, 131 S. Loomis, St., Naperville IL 60540, USA; b Department of Chemistry, Illinois Institute of Technology, 3101 S. Dearborn St., Chicago IL 60616, USA

**Keywords:** powder diffraction, citrate, calcium, Rietveld refinement, density functional theory

## Abstract

The crystal structure of ‘aquabis­(di­hydrogen citrato)calcium hydrate’, better formulated as aqua­(citric acid)(hydrogen citrato)calcium monohydrate, (I), has been solved and refined using synchrotron X-ray powder diffraction data, and optimized using density functional techniques. The CaO_8_ coordination polyhedra are isolated, but occur in layers parallel to the *ab* plane. Both the Rietveld-refined and DFT-optimized structures indicate that one citrate is doubly ionized and that the other is citric acid. All of the active hydrogen atoms participate in strong (11–16 kcal mol^−1^) hydrogen bonds. Hydrogen atoms were added to the existing calcium hydrogen citrate trihydrate structure [Sheldrick (1974 ▸). *Acta Cryst.* B**30**, 2056–2057; CSD refcode: CAHCIT], (II), and a DFT calculation was carried out to assess the hydrogen bonding and compare it to this optimized structure.

## Abstract 2

The crystal structure of aqua­(citric acid)(hydrogen citrato)calcium monohydrate has been solved and refined using synchrotron X-ray powder diffraction data, and optimized using density functional techniques. A DFT-optimized structure is also reported for calcium hydrogen citrate trihydrate.

## Chemical context   

A systematic study of the crystal structures of Group 1 (alkali metal) citrate salts has been reported in Rammohan & Kaduk (2018 ▸). This paper is part of an extension of the study to Group 2 (alkaline earth) citrates. Calcium citrate (as the tetra­hydrate) is a common dietary supplement. Previously reported calcium citrate structures include calcium hydrogen citrate trihydrate (Sheldrick, 1974 ▸; CSD refcode: CAHCIT) and calcium citrate tetra­hydrate (Herdtweck *et al.*, 2011 ▸; ISEQIH). The ISEQIH structure was derived from a hydro­thermally synthesized pseudomerohedrally twinned crystal at 123 K, and in this study a Rietveld refinement using room-temperature powder data was also reported.
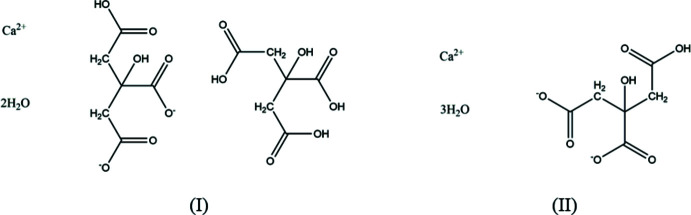


The crystal structures of anhydrous calcium citrate, a new (and commercially relevant, for it is the phase that occurs in dietary supplements) polymorph of calcium citrate tetra­hydrate, and calcium citrate hexa­hydrate have been reported recently (Kaduk, 2018 ▸).

## Structural commentary   

**Figure 1:**
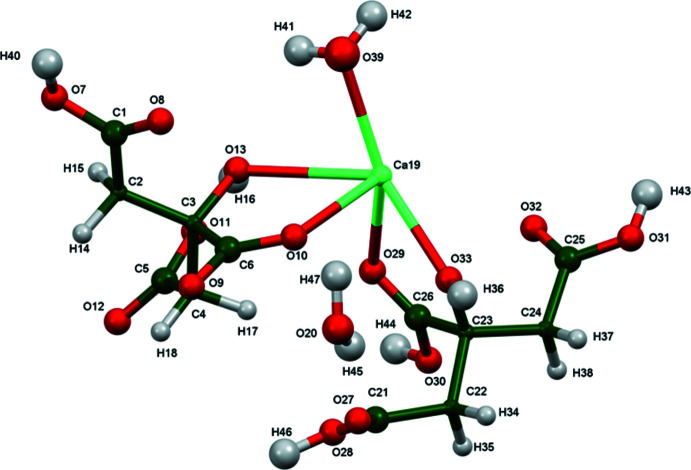
The asymmetric unit of aqua­(citric acid)(hydrogen citrato)calcium monohydrate with the atom numbering and 50% probability spheroids.

**Figure 2:**
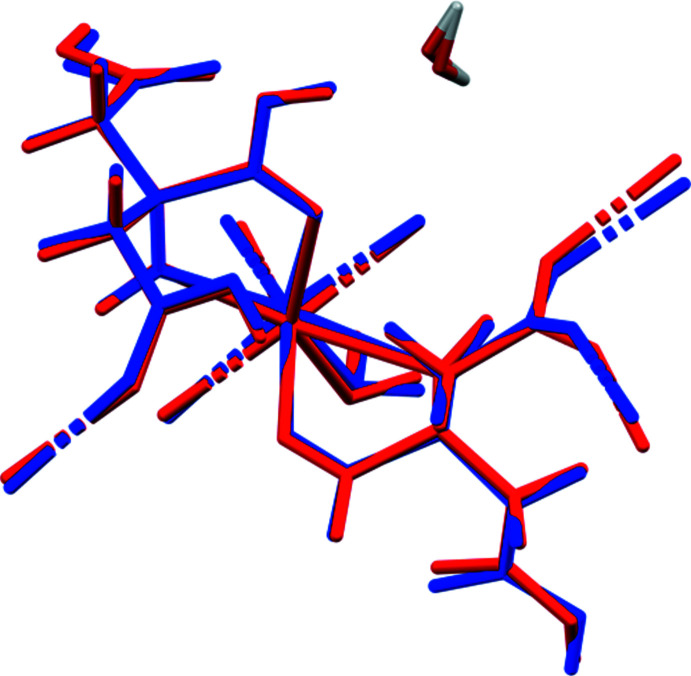
Comparison of the refined and optimized structures of aqua­(citric acid)(hydrogen citrato)calcium monohydrate. The refined structure is in red, and the DFT-optimized structure is in blue.

The crystal structure of ‘aquabis­(di­hydrogen citrato)calcium hydrate’, (I), has been solved and refined using synchrotron X-ray powder diffraction data, and optimized using density functional techniques (Fig. 1 ▸). The root-mean-square Cartesian displacement of the non-hydrogen citrate atoms in the Rietveld refined and DFT-optimized structures is 0.213 Å (Fig. 2 ▸) The absolute difference in the position of the Ca^2+^ cation in the unit cell is 0.318 Å. The good agreement between the structures is evidence that the experimental structure is correct (van de Streek & Neumann, 2014 ▸). The rest of the discussion will emphasize the DFT-optimized structure. Almost all of the citrate bond lengths, bond angles, and torsion angles fall within the normal ranges indicated by a *Mercury* Mogul geometry check (Macrae *et al.*, 2020 ▸). Only the C4—C3—C6 angle of 102.6° [average = 110.4 (19), *Z*-score = 4. 2] is flagged as unusual. One citrate occurs in the *trans,trans*-conformation, and the other occurs in the *gauche*,*trans-*conformation. Both conformations are equivalent in energy (Rammohan & Kaduk, 2018 ▸). Both central carboxyl­ate groups and the hydroxyl groups exhibit slight twists (O10—C6—C3—O13 = −18.1 and O29—C26—C23—O33 = −4.1°) from the normal planar arrangement.

Both the refined and optimized structures indicate that one citrate group (C1–C6) is doubly ionized (in the normal fashion central/terminal), with the C1—O8—O7—H40 moiety being an intact carb­oxy­lic acid group. The other ‘citrate’ (C21–C26) is in fact citric acid. The compound may therefore be better characterized as aqua­(citric acid)(hydrogen citrato)calcium monohydrate. The C—O bond lengths in both the refined (restrained) and optimized structures are consistent with this formulation. Removing the restraints on the C—O bond lengths did not change the refined values significantly. Given the preparation (in a probable excess of citric acid), the crystallization of a mixed salt/co-crystal is not unreasonable. It is probably wise to be cautious about locating hydrogen atoms using X-ray powder (even synchrotron) data, even when the structure is confirmed by a DFT calculation. As noted below, some of the hydrogen bonds are very strong, and perhaps have double minima. A more sophisticated quantum calculation may be required to understand the details of the hydrogen bonding in this compound.

The Ca^2+^ cation is eight-coordinate, with seven shorter and one long bond (Table 1 ▸), resulting in a distorted bicapped octa­hedral coordination polyhedron; the ligands are two terminal carboxyl­ate groups, one central carboxyl­ate group, one terminal CO_2_H, one central CO_2_H, two hydroxyl groups, and one water mol­ecule. The Ca bond-valence sum amounts to 2.10 valence units (v.u.). Both citrate and citric acid chelate to the Ca^2+^ cation through the hydroxyl groups and the central carboxyl groups (O13/O10 and O33/O29), respectively, forming a five-membered ring. The citrate anion also exhibits a monodentate mode to two other Ca^2+^ cations (through O11 and O12) whereas the citric acid mol­ecule shows a monodentate coordination mode only through O27.

The Bravais–Friedel–Donnay–Harker (Bravais, 1866 ▸; Friedel, 1907 ▸; Donnay & Harker, 1937 ▸) method suggests that we might expect blocky morphology for aqua­(citric acid)(hydrogen citrato)calcium monohydrate. A 2nd order spherical harmonic model was included in the refinement. The texture index was only 1.003, indicating that preferred orientation was not significant in this rotated capillary specimen.

In the known crystal structure of CAHCIT, (II), the citrate anion is also in the *gauche*,*trans*-conformation, and chelates to the Ca^2+^ cation through the hydroxyl group and a terminal carboxyl­ate group as well as through the ionized central and terminal carboxyl­ate groups. The root-mean-square Cartesian displacement between the single crystal and the DFT-optimized structures is only 0.0399 Å, confirming the excellent quality of the single-crystal study (Sheldrick, 1974 ▸). The Ca bond-valence sum is 2.07 v.u. With a limited number of calcium citrate structures, it is hard to make grand generalizations, but several more such compounds have been synthesized and await structural characterization.

## Supra­molecular features   

**Figure 3:**
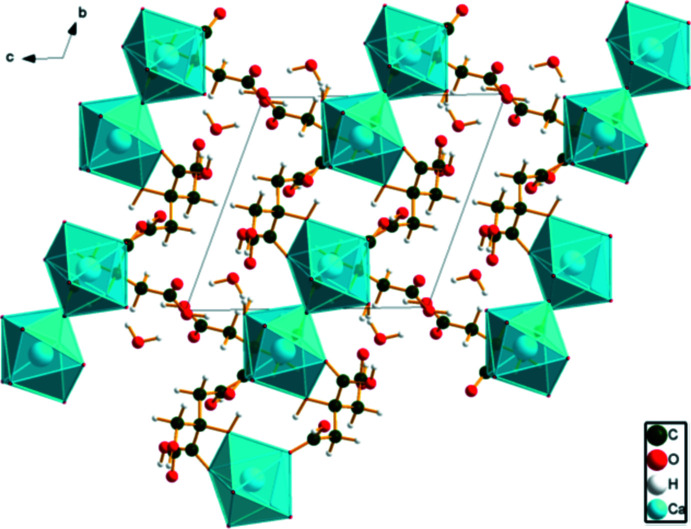
Crystal structure of aqua­(citric acid)(hydrogen citrato)calcium monohydrate, viewed down the *a* axis.

The CaO_8_ coordination polyhedra in aqua­(citric acid)(hydrogen citrato)calcium monohydrate are isolated (Fig. 3 ▸), but occur in layers parallel to the *ab* plane. Numerical values of the hydrogen bonds are summarized in Table 2 ▸. The free water mol­ecule O20 acts as a hydrogen-bond donor to the hydroxyl group O13 and the carbonyl group O8. The coordinating water mol­ecule O39 acts as a donor to the carbonyl group O32 and the free water mol­ecule O20. The carb­oxy­lic acid group O7—H40 in the hydrogen citrate anion acts as a donor to the coordinating water mol­ecule O39. The carb­oxy­lic acid function O28—H46 acts as a donor to the ionized central carboxyl­ate O9. The carb­oxy­lic acids O31—H43 and O30—H44 act as donors to the free water mol­ecule O20. The hydroxyl group O13—H16 forms an intra­molecular hydrogen bond to the ionized central carboxyl­ate O11 atom, while the hydroxyl group O33—H36 forms an inter­molecular hydrogen bond to the ionized central carboxyl­ate O10 atom.

**Figure 4:**
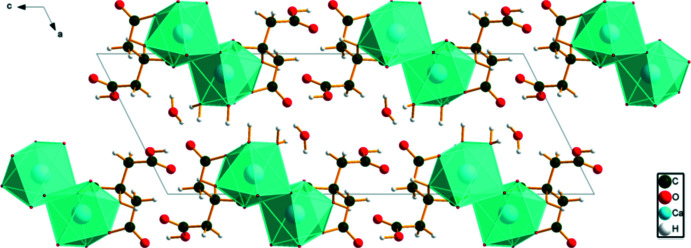
Crystal structure of [Ca(C_6_H_6_O_7_(H_2_O)_3_], viewed down the *b* axis.

In CAHCIT, the Ca^2+^ cation is seven-coordinated in the form of a distorted side-capped trigonal prism. The polyhedra share corners to form chains along the [010] direction. In Ca_3_(C_6_H_5_O_7_)_2_ and its hydrates (Kaduk, 2018 ▸), the coordination numbers are larger, and the Ca/O coordination spheres share edges to form layers, which condense to a three-dimensional framework in anhydrous calcium citrate. Since the hydrogen atoms were not located in the CAHCIT structure, approximate positions were deduced, and a DFT calculation was carried out to assess the hydrogen bonding (Table 3 ▸). The carb­oxy­lic acid function O13—H23 acts as a donor to the ionized carboxyl­ate O8 atom. The hydroxyl group O9—H20 forms an inter­molecular hydrogen bond to the carbonyl O12 atom. The water mol­ecules act as donors to both ionized carboxyl­ate groups and other water mol­ecules. The crystal structure of calcium hydrogen citrate trihydrate is shown in Fig. 4 ▸.

## Database survey   

Details of the comprehensive literature search for citrate structures are presented in Rammohan & Kaduk (2018 ▸). A search of the Cambridge Structural Database (Groom *et al.*, 2016 ▸, version 2020.2.0) using a citrate fragment and Ca, C, H, and O only yielded two hits, *viz*. calcium hydrogen citrate trihydrate (Sheldrick, 1974 ▸; CAHCIT) and calcium citrate tetra­hydrate (Herdtweck *et al.*, 2011 ▸; ISEQIH). A search of the Powder Diffraction File (Gates-Rector & Blanton, 2019 ▸) for C, H, Ca, and O only with ‘citrat’ in the compound name yielded entry 00-028-2003 for the mineral earlandite (calcium citrate tetra­hydrate, isolated from an unconsolidated ocean floor sediment from the Weddell Sea near Antarctica), 00-069-1272, 1273, and 1274 for the three compounds from Kaduk (2018 ▸), 01-084-5956 calculated from ISEQIH, and 02-060-8946 calculated from CAHCIT.

## Synthesis and crystallization   

This solid was obtained from the scale [94.5 (1) wt% magnesian calcite Ca_0.84_Mg_0.16_CO_3_, 5.3 (4) wt% brucite Mg(OH)_2_, and 0.2 (1) wt% vaterite polymorph of CaCO_3_] in a Megahome water still. The still was cleaned by filling the tank with tap water (from Lake Michigan; 47 ppm Ca and 11 ppm Mg), adding several tablespoons of citric acid monohydrate, and boiling for ∼2 h. The pale-yellow solution was deca­nted into a plastic pail, and allowed to evaporate at ambient conditions. This solid was recovered after 90 days, with isolation of inter­mediate phases. The wet solid was washed with ethanol to remove the yellow syrup from the white solid. The slurry was filtered and dried in an oven at 338 K. The solid was first ground in a mortar and pestle, then in a Spex 8000 mixer/mill.

## Refinement   

The laboratory pattern (measured on a Bruker D2 Phaser using Cu radiation) was indexed on a primitive triclinic unit cell using *DICVOL06* (Louër & Boultif, 2007 ▸). The indexing was carried out on a pattern from a specimen blended with NIST SRM 640b Si inter­nal standard; *a* = 8.37261 (3), *b* = 10.90306 (4), *c* = 11.06287 (4) Å, *α* = 105.2026 (4), *β* = 100.6846 (4), *γ* = 110.7096 (3)°, *V* = 867.2026 (4) Å^3^, and *Z* = 2. This cell was used to solve the structure with data collected from beamline 11-BM (Lee *et al.*, 2008 ▸; Wang *et al.*, 2008 ▸) at the Advanced Photon Source, Argonne National Laboratory using direct methods in *EXPO2009* (Altomare *et al.*, 2009 ▸).

**Figure 5:**
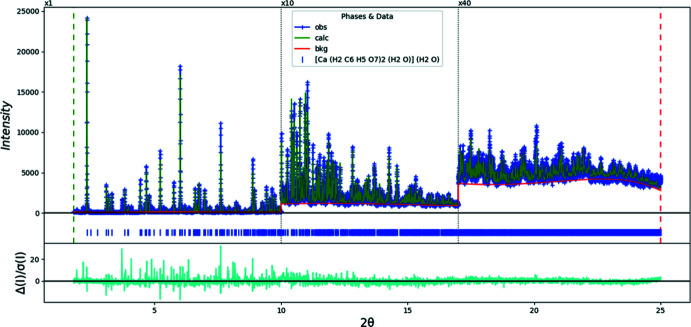
Rietveld plot for aqua­(citric acid)(hydrogen citrato)calcium monohydrate. The blue crosses represent the observed data points, and the green line is the calculated pattern. The cyan curve is the normalized error plot. The vertical scale has been multiplied by a factor of 10× for 2*θ* > 10.0°, and by a factor of 40× for 2*θ* > 17.0°. The row of blue tick marks indicates the calculated reflection positions; the red line is the background curve.

Crystal data, data collection and structure refinement details are summarized in Table 4 ▸. The structure was refined by the Rietveld method using *GSAS-II* (Toby & Von Dreele, 2013 ▸). Initial positions for the active hydrogen atoms were derived by an analysis of potential hydrogen-bonding patterns. All non-H bond lengths and angles in the citrate anion/citric acid mol­ecule were subjected to restraints, based on a *Mercury* Mogul geometry check (Sykes *et al.*, 2011 ▸; Bruno *et al.*, 2004 ▸) of the mol­ecule; the Ca—O bond lengths were not restrained. The Mogul average and standard deviation for each qu­antity were used as the restraint parameters. The hydrogen atoms were included in calculated positions, which were recalculated during the refinement using *Materials Studio* (Dassault Systèmes, 2018 ▸). *U*_iso_ values were grouped by chemical similarity, and the *U*_iso_ value in the two anions were constrained to be the same. The *U*_iso_ value of each H atom was constrained to be 1.3× that of the heavy atom to which is is attached. A Rietveld plot is given in Fig. 5 ▸. The largest errors in the difference plot reflect the presence of an unidentified impurity and misfits of the peak profiles. The peaks of the impurity phase can be indexed on a primitive monoclinic unit cell with *a* = 15.3648, *b* = 7.2713, *c* = 19.3755 Å, *β* = 109.116°, and *V* = 2045.30 Å^3^, but no structure solution has as yet been obtained.

Density functional geometry optimizations (fixed experimental unit cell) for the two structures were carried out using *CRYSTAL09* (Dovesi *et al.*, 2005 ▸). The basis sets for the H, C and O atoms were those of Gatti *et al.* (1994 ▸), and the basis set for Ca was that of Catti *et al.* (1991 ▸). The calculations used 8 *k*-points and the B3LYP functional, and each took around seven days on a 2.4 GHz PC.

## Supplementary Material

Crystal structure: contains datablock(s) I, I_DFT, CAHCIT_DFT. DOI: 10.1107/S2056989020012864/wm5577sup1.cif

Structure factors: contains datablock(s) I. DOI: 10.1107/S2056989020012864/wm5577Isup2.hkl

Rietveld powder data: contains datablock(s) I. DOI: 10.1107/S2056989020012864/wm5577Isup3.rtv

CCDC references: 2033184, 2033183, 2033182

Additional supporting information:  crystallographic information; 3D view; checkCIF report

## Figures and Tables

**Table 1:** Selected bond lengths (Å) for (I)

Ca19—O12^i^	2.332	Ca19—O29	2.446
Ca19—O33	2.400	Ca19—O39	2.466
Ca19—O11^ii^	2.417	Ca19—O27^iii^	2.564
Ca19—O10	2.421	Ca19—O13	2.818

Symmetry codes: (i) 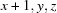; (ii) 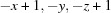; (iii) 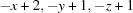.

**Table 2:** Hydrogen-bond geometry (Å, °, electrons, kcal mol^−1^) for (I)

*D*—H⋯*A*	*D*—H	H⋯*A*	*D*⋯*A*	*D*—H⋯*A*	Mulliken overlap	H-bond energy
O28^i^—H46⋯O9	1.022	1.542	2.545	165.8	0.087	16.1
O20—H47⋯O13^ii^	0.999	1.726	2.706	166.0	0.078	15.3
O7—H40⋯O39^iii^	1.001	1.706	2.677	162.4	0.076	15.1
O33—H36⋯O10^iv^	1.002	1.683	2.672	168.4	0.070	14.5
O30—H44⋯O20	0.999	1.687	2.685	178.1	0.066	14.0
O13—H16⋯O11	0.995	1.852	2.739	146.8	0.061	13.5
O20—H45⋯O8^v^	0.991	1.798	2.754	161.0	0.059	13.3
O39—H41⋯O20^ii^	0.978	1.815	2.765	163.2	0.057	13.0
O39—H42⋯O32^vi^	0.986	1.816	2.739	154.3	0.049	12.1
O31—H43⋯O20^vii^	0.984	1.816	2.851	159.6	0.042	11.2

Symmetry codes: (i) 1 − *x*, 1 − *y*, 1 − *z*; (ii) 1 − *x*, −*y*, 1 − *z*; (iii) 1 − *x*, −*y*, −*z*; (iv) 2 − *x*, 1 − *y*, 1 − *z*; (v) *x*, *y*, 1 + *z*; (vi) 2 − *x*, −*y*, 1 − *z*; (vii) 1 + *x*, *y*, *z*.

**Table 3:** Hydrogen-bond geometry (Å, °, electrons, kcal mol^−1^) for (II)

*D*—H⋯*A*	*D*—H	H⋯*A*	*D*⋯*A*	*D*—H⋯*A*	Mulliken overlap	H-bond energy
O15^i^—H29⋯O8^ii^	0.974	1.940	2.891	164.6	0.035	10.2
O15^iii^—H28⋯O10^iv^	0.979	1.831	2.806	173.5	0.046	11.7
O16^i^—H27⋯O7^vi^	0.971	1.970	2.926	167.6	0.037	10.5
O16^i^—H26⋯O7^i^	0.981	1.838	2.817	176.3	0.055	12.8
O14^v^—H25⋯O15^i^	0.992	1.719	2.702	170.9	0.081	15.6
O14^v^—H24⋯O8^v^	0.975	1.782	2.742	167.1	0.047	11.8
O13^v^—H23⋯O8^v^	1.018	1.560	2.571	171.4	0.076	15.1
O9^v^—H20⋯O12^i^	0.988	1.764	2.749	174.9	0.061	13.5

Symmetry codes: (i) *x*, 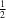 − *y*, 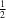 + *z*; (ii) 1 − *x*, −*y*, 1 − *z*; (iii) 1 − *x*, 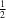 + *y*, 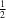 − *z*; (iv) *x*, 1 + *y*, *z*; (v) 1 − *x*, 1 − *y*, 1 − *z*.

**Table 4:** Experimental details

	(I)
Crystal data	
Chemical formula	[Ca(C_6_H_6_O_7_)(C_6_H_8_O_7_)(H_2_O)]·H_2_O
*M*_r_	458.34
Crystal system, space group	Triclinic, *P* 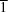
Temperature (K)	295
*a*, *b*, *c* (Å)	8.37267 (11), 10.9032 (3), 11.0629 (3)
α, β, γ (°)	105.2029 (6), 100.6847 (4), 110.7096 (3)
*V* (Å^3^)	867.24 (1)
*Z*	2
Radiation type	Synchrotron, λ = 0.41307 Å
Specimen shape, size (mm)	Cylinder, 3 × 1.5

Data collection	
Diffractometer	11-BM, APS
Specimen mounting	Kapton capillary
Data collection mode	Transmission
Scan method	Step
2θ values (°)	2θ_min_ = 0.500, 2θ_max_ = 49.991, 2θ_step_ = 0.001

Refinement	
*R* factors and goodness of fit	*R*_p_ = 0.116, *R*_wp_ = 0.154, *R*_exp_ = 0.066, *R*(*F* ^2^) = 0.11717, χ^2^ = 5.532
No. of parameters	115
No. of restraints	68
H-atom treatment	Only H-atom displacement parameters refined
(Δ/σ)_max_	0.163

Computer programs: *EXPO2009* (Altomare *et al.*, 2009 ▸), *GSAS-II* (Toby & Von Dreele, 2013 ▸), *Mercury* (Macrae *et al.*, 2020 ▸), *DIAMOND* (Brandenburg, 2016 ▸) and *publCIF* (Westrip, 2010 ▸).

## supplementary crystallographic information

### Aqua(citric acid)(hydrogen citrato)calcium monohydrate (I) Crystal data

**Table:**

[Ca(C_6_H_6_O_7_)(C_6_H_8_O_7_)(H_2_O)]·H_2_O	*V* = 867.24 (1) Å^3^
*M**_r_* = 458.34	*Z* = 2
Triclinic, *P*1	*D*_x_ = 1.755 Mg m^−^^3^
Hall symbol: -P 1	Synchrotron radiation, λ = 0.41307 Å
*a* = 8.37267 (11) Å	*T* = 295 K
*b* = 10.9032 (3) Å	Particle morphology: white powder
*c* = 11.0629 (3) Å	white
α = 105.2029 (6)°	cylinder, 3 × 1.5 mm
β = 100.6847 (4)°	Specimen preparation: Prepared at 300 K
γ = 110.7096 (3)°	

### Aqua(citric acid)(hydrogen citrato)calcium monohydrate (I) Data collection

**Table:**

11-BM, APS diffractometer	Scan method: step
Specimen mounting: Kapton capillary	2θ_min_ = 0.500°, 2θ_max_ = 49.991°, 2θ_step_ = 0.001°
Data collection mode: transmission	

### Aqua(citric acid)(hydrogen citrato)calcium monohydrate (I) Refinement

**Table:**

Least-squares matrix: full	115 parameters
*R*_p_ = 0.116	68 restraints
*R*_wp_ = 0.154	Only H-atom displacement parameters refined
*R*_exp_ = 0.066	Weighting scheme based on measured s.u.'s
*R*(*F*^2^) = 0.11717	(Δ/σ)_max_ = 0.163
49492 data points	Background function: Background function: "chebyschev-1" function with 6 terms: 86.9(17), 10.6(26), -9.1(12), -14.2(8), 19.3(10), -23.7(7), Background peak parameters: pos, int, sig, gam: 5.20(3), 5.0(4)e5, 3.10(18)e4, 0.100,
Profile function: Finger-Cox-Jephcoat function parameters U, V, W, X, Y, SH/L: peak variance(Gauss) = Utan(Th)^2^+Vtan(Th)+W: peak HW(Lorentz) = X/cos(Th)+Ytan(Th); SH/L = S/L+H/L U, V, W in (centideg)^2^, X & Y in centideg 1.163, -0.126, 0.063, 0.000, 0.000, 0.002, Crystallite size in microns with "isotropic" model: parameters: Size, G/L mix 1.41(5), 1.000, Microstrain, "isotropic" model (10^6^ * delta Q/Q) parameters: Mustrain, G/L mix 1026(17), 1.000,	Preferred orientation correction: Simple spherical harmonic correction Order = 2 Coefficients: 0:0:C(2,-2) = 0.041(5); 0:0:C(2,-1) = 0.002(5); 0:0:C(2,0) = -0.053(5); 0:0:C(2,1) = -0.047(5); 0:0:C(2,2) = 0.076(5)

### Aqua(citric acid)(hydrogen citrato)calcium monohydrate (I) Fractional atomic coordinates and isotropic or equivalent isotropic displacement parameters (Å^2^)

**Table:**

	*x*	*y*	*z*	*U*_iso_*/*U*_eq_	
C1	0.3002 (7)	0.0929 (11)	0.1064 (5)	0.0317 (7)*	
C2	0.2629 (8)	0.1243 (8)	0.2378 (6)	0.0194 (16)*	
C3	0.4299 (6)	0.1958 (4)	0.3587 (4)	0.0194*	
C4	0.3777 (8)	0.2398 (5)	0.4829 (6)	0.0194*	
C5	0.2425 (8)	0.1186 (7)	0.5031 (8)	0.0317*	
C6	0.5717 (6)	0.3282 (6)	0.3500 (8)	0.0317*	
O7	0.1573 (8)	0.0272 (7)	0.0020 (6)	0.0317*	
O8	0.4552 (7)	0.1313 (7)	0.0985 (6)	0.0317*	
O9	0.5212 (8)	0.4142 (6)	0.3257 (7)	0.0317*	
O10	0.7340 (7)	0.3519 (6)	0.3923 (7)	0.0317*	
O11	0.2937 (8)	0.0305 (7)	0.5283 (7)	0.0317*	
O12	0.0867 (8)	0.1091 (6)	0.4861 (7)	0.0317*	
O13	0.5123 (7)	0.1008 (6)	0.3667 (6)	0.0317*	
H14	0.19243	0.179707	0.214454	0.0252*	
H15	0.16994	0.02156	0.24318	0.0252*	
H16	0.46014	0.04456	0.40231	0.0412*	
H17	0.50220	0.289194	0.57285	0.0252*	
H18	0.31942	0.31872	0.47649	0.0252*	
Ca19	0.8557 (3)	0.2040 (3)	0.4459 (3)	0.0265 (10)*	
O20	0.5714 (9)	0.1614 (7)	0.8826 (7)	0.034 (3)*	
C21	0.9040 (8)	0.6012 (10)	0.7264 (9)	0.0317*	
C22	1.0577 (9)	0.5859 (7)	0.8073 (6)	0.0194*	
C23	1.0641 (5)	0.4425 (5)	0.7610 (4)	0.0194*	
C24	1.2319 (8)	0.4523 (7)	0.8537 (6)	0.0194*	
C25	1.2572 (8)	0.3207 (6)	0.8292 (11)	0.0317*	
C26	0.8915 (7)	0.3260 (7)	0.7618 (5)	0.0317*	
O27	0.9197 (8)	0.6604 (7)	0.6442 (7)	0.0317*	
O28	0.7536 (8)	0.5408 (7)	0.7496 (7)	0.0317*	
O29	0.7793 (8)	0.2414 (7)	0.6568 (5)	0.0317*	
O30	0.8722 (8)	0.3353 (7)	0.8750 (6)	0.0317*	
O31	1.4295 (8)	0.3460 (6)	0.8536 (7)	0.0317*	
O32	1.1330 (8)	0.2043 (6)	0.7770 (7)	0.0317*	
O33	1.0718 (7)	0.4071 (6)	0.6278 (5)	0.0317*	
H34	1.19050	0.66534	0.810791	0.0252*	
H35	1.05265	0.61039	0.91286	0.0252*	
H36	1.12701	0.48610	0.62146	0.0412*	
H37	1.35653	0.53313	0.854557	0.0252*	
H38	1.22758	0.48943	0.95894	0.0252*	
O39	0.8031 (10)	0.0427 (8)	0.2335 (7)	0.051 (3)*	
H40	0.18003	−0.01867	−0.07989	0.0412*	
H41	0.68916	−0.03767	0.23211	0.0663*	
H42	0.85248	−0.02514	0.24834	0.0663*	
H43	1.43719	0.26928	0.82734	0.0412*	
H44	0.74472	0.27820	0.86966	0.0412*	
H45	0.55021	0.11781	0.94379	0.0441*	
H46	0.65968	0.57637	0.77733	0.0412*	
H47	0.55075	0.08274	0.80181	0.0441*	

### Aqua(citric acid)(hydrogen citrato)calcium monohydrate (I) Geometric parameters (Å, º)

**Table:**

C1—C2	1.519 (6)	Ca19—O27^iv^	2.494 (6)
C1—O7	1.314 (6)	Ca19—O29	2.501 (6)
C1—O8	1.243 (6)	Ca19—O33	2.388 (6)
C2—C1	1.519 (6)	Ca19—O39	2.389 (7)
C2—C3	1.530 (4)	C21—C22	1.512 (6)
C3—C2	1.530 (4)	C21—O27	1.246 (6)
C3—C4	1.527 (4)	C21—O28	1.306 (5)
C3—C6	1.551 (3)	C22—C21	1.512 (6)
C3—O13	1.445 (3)	C22—C23	1.536 (4)
C4—C3	1.527 (4)	C23—C22	1.536 (4)
C4—C5	1.507 (4)	C23—C24	1.529 (4)
C5—C4	1.507 (4)	C23—C26	1.554 (3)
C5—O11	1.253 (5)	C23—O33	1.442 (3)
C5—O12	1.246 (5)	C24—C23	1.529 (4)
C6—C3	1.551 (3)	C24—C25	1.486 (6)
C6—O9	1.225 (4)	C25—C24	1.486 (6)
C6—O10	1.259 (4)	C25—O31	1.328 (6)
O7—C1	1.314 (6)	C25—O32	1.219 (6)
O8—C1	1.243 (6)	C26—C23	1.554 (3)
O9—C6	1.225 (4)	C26—O29	1.226 (5)
O10—C6	1.259 (4)	C26—O30	1.275 (6)
O10—Ca19	2.330 (6)	O27—Ca19^iv^	2.494 (6)
O11—C5	1.253 (5)	O27—C21	1.246 (6)
O11—Ca19^i^	2.537 (6)	O28—C21	1.306 (5)
O12—C5	1.246 (5)	O29—Ca19	2.501 (6)
O12—Ca19^ii^	2.519 (6)	O29—C26	1.226 (5)
O13—C3	1.445 (3)	O30—C26	1.275 (6)
O13—Ca19	2.562 (6)	O31—C25	1.328 (6)
Ca19—O10	2.330 (6)	O32—C25	1.219 (6)
Ca19—O11^i^	2.537 (6)	O33—Ca19	2.388 (6)
Ca19—O12^iii^	2.519 (6)	O33—C23	1.442 (3)
Ca19—O13	2.562 (6)	O39—Ca19	2.389 (7)
			
C2—C1—O7	115.3 (4)	O33—Ca19—O39	145.5 (2)
C2—C1—O8	122.2 (4)	C22—C21—O27	123.3 (5)
O7—C1—O8	122.5 (4)	C22—C21—O28	112.1 (5)
C1—C2—C3	114.8 (4)	O27—C21—O28	124.6 (4)
C2—C3—C4	109.7 (3)	C21—C22—C23	116.7 (6)
C2—C3—C6	111.3 (3)	C22—C23—C24	107.8 (3)
C4—C3—C6	108.6 (3)	C22—C23—C26	110.2 (4)
C2—C3—O13	109.8 (2)	C24—C23—C26	111.1 (3)
C4—C3—O13	110.13 (19)	C22—C23—O33	110.3 (2)
C6—C3—O13	107.4 (3)	C24—C23—O33	110.1 (2)
C3—C4—C5	113.3 (3)	C26—C23—O33	107.4 (3)
C4—C5—O11	116.9 (5)	C23—C24—C25	116.0 (5)
C4—C5—O12	117.8 (4)	C24—C25—O31	111.7 (4)
O11—C5—O12	125.1 (4)	C24—C25—O32	123.0 (5)
C3—C6—O9	117.5 (3)	O31—C25—O32	124.6 (4)
C3—C6—O10	117.1 (2)	C23—C26—O29	119.2 (3)
O9—C6—O10	124.0 (4)	C23—C26—O30	115.3 (3)
C6—O10—Ca19	129.0 (3)	O29—C26—O30	125.10 (19)
O10—Ca19—O33	86.2 (2)	Ca19—O33—C23	125.0 (3)
O10—Ca19—O39	101.9 (3)		

Symmetry codes: (i) −*x*+1, −*y*, −*z*+1; (ii) *x*−1, *y*, *z*; (iii) *x*+1, *y*, *z*; (iv) −*x*+2, −*y*+1, −*z*+1.

### (I_DFT) Crystal data

**Table:**

C_12_H_18_CaO_16_	*c* = 11.0629 Å
*M**_r_* = 458.34	α = 105.2026°
Triclinic, *P*1	β = 100.6846°
Hall symbol: -P 1	γ = 110.7096°
*a* = 8.3726 Å	*V* = 867.24 Å^3^
*b* = 10.9031 Å	*Z* = 2

### (I_DFT) Data collection

**Table:**

*h* = →	*l* = →
*k* = →	

### (I_DFT) Fractional atomic coordinates and isotropic or equivalent isotropic displacement parameters (Å^2^)

**Table:**

	*x*	*y*	*z*	*U*_iso_*/*U*_eq_	
C1	0.31016	0.07285	0.08749	0.02690*	
C2	0.26956	0.10747	0.21719	0.01140*	
C3	0.43277	0.17813	0.34179	0.01140*	
C4	0.38652	0.23372	0.46820	0.01140*	
C5	0.25177	0.12171	0.50135	0.02690*	
C6	0.57901	0.31142	0.34106	0.02690*	
O7	0.16469	−0.01796	−0.01141	0.02690*	
O8	0.46026	0.12456	0.07496	0.02690*	
O9	0.53090	0.39795	0.30853	0.02690*	
O10	0.74165	0.33221	0.38382	0.02690*	
O11	0.28897	0.01869	0.50683	0.02690*	
O12	0.11984	0.13979	0.52504	0.02690*	
O13	0.50583	0.07864	0.35311	0.02690*	
H14	0.19948	0.17489	0.21364	0.01480*	
H15	0.17267	0.01068	0.22038	0.01480*	
H16	0.44873	0.03225	0.41011	0.03500*	
H17	0.51107	0.28420	0.55055	0.01480*	
H18	0.33695	0.31178	0.46016	0.01480*	
Ca19	0.88141	0.19783	0.46615	0.01810*	
O20	0.54811	0.14774	0.85153	0.03720*	
C21	0.89574	0.61406	0.72810	0.02690*	
C22	1.05088	0.60366	0.81326	0.01140*	
C23	1.05835	0.45929	0.76591	0.01140*	
C24	1.22583	0.46859	0.86206	0.01140*	
C25	1.24934	0.33401	0.83148	0.02690*	
C26	0.88489	0.34266	0.76154	0.02690*	
O27	0.91283	0.67660	0.65013	0.02690*	
O28	0.74438	0.55486	0.75249	0.02690*	
O29	0.77908	0.24851	0.65899	0.02690*	
O30	0.85938	0.35541	0.87736	0.02690*	
O31	1.41952	0.35627	0.84442	0.02690*	
O32	1.12639	0.21838	0.80356	0.02690*	
O33	1.06496	0.42342	0.63541	0.02690*	
H34	1.17517	0.68282	0.81372	0.01480*	
H35	1.03899	0.62482	0.91229	0.01480*	
H36	1.14234	0.50804	0.61969	0.03500*	
H37	1.34467	0.55122	0.85978	0.01480*	
H38	1.21569	0.49440	0.96204	0.01480*	
O39	0.81696	0.02002	0.25093	0.04720*	
H40	0.18537	−0.02945	−0.09914	0.04120*	
H41	0.68916	−0.03767	0.23211	0.06630*	
H42	0.86902	−0.04787	0.25331	0.06630*	
H43	1.43590	0.26928	0.83176	0.04120*	
H44	0.74472	0.27820	0.86966	0.04120*	
H45	0.53070	0.12674	0.93125	0.04410*	
H46	0.35770	0.42024	0.28145	0.04120*	
H47	0.54619	0.06538	0.78398	0.04410*	

### (I_DFT) Bond lengths (Å)

**Table:**

C1—C2	1.518	Ca19—O13	2.818
C1—O7	1.321	O20—H45	0.991
C1—O8	1.233	O20—H47	0.999
C2—C3	1.537	C21—C22	1.515
C2—H14	1.094	C21—O27	1.231
C2—H15	1.094	C21—O28	1.317
C3—C4	1.548	C22—C23	1.551
C3—C6	1.537	C22—H34	1.090
C3—O13	1.442	C22—H35	1.090
C4—C5	1.521	C23—C24	1.548
C4—H17	1.095	C23—C26	1.538
C4—H18	1.086	C23—O33	1.410
C5—O11	1.280	C24—C25	1.508
C5—O12	1.250	C24—H37	1.091
C6—O9	1.255	C24—H38	1.095
C6—O10	1.271	C25—O31	1.331
O7—H40	1.001	C25—O32	1.227
O11—Ca19^i^	2.417	C26—O29	1.224
O12—Ca19^ii^	2.332	C26—O30	1.317
O13—H16	0.995	O27—Ca19^iv^	2.564
Ca19—O12^iii^	2.332	O28—H46^v^	1.022
Ca19—O33	2.400	O30—H44	0.999
Ca19—O11^i^	2.417	O31—H43	0.984
Ca19—O10	2.421	O33—H36	1.002
Ca19—O29	2.446	O39—H41	0.978
Ca19—O39	2.466	O39—H42	0.986
Ca19—O27^iv^	2.564	H46—O28^v^	1.022

Symmetry codes: (i) −*x*+1, −*y*, −*z*+1; (ii) *x*−1, *y*, *z*; (iii) *x*+1, *y*, *z*; (iv) −*x*+2, −*y*+1, −*z*+1; (v) −*x*+1, −*y*+1, −*z*+1.

### (I_DFT) Hydrogen-bond geometry (Å, º)

**Table:**

*D*—H···*A*	*D*—H	H···*A*	*D*···*A*	*D*—H···*A*
O28^v^—H46···O9	1.022	1.542	2.545	165.8
O20—H47···O13^i^	0.999	1.726	2.706	166.0
O7—H40···O39^vi^	1.001	1.706	2.677	162.4
O33—H36···O10^iv^	1.002	1.683	2.672	168.4
O30—H44···O20	0.999	1.687	2.685	178.1
O13—H16···O11	0.995	1.852	2.739	146.8
O20—H45···O8^vii^	0.991	1.798	2.754	161.0
O39—H41···O20^i^	0.978	1.815	2.765	163.2
O39—H42···O32^viii^	0.986	1.816	2.739	154.3
O31—H43···O20^iii^	0.984	1.816	2.851	159.6

Symmetry codes: (i) −*x*+1, −*y*, −*z*+1; (iii) *x*+1, *y*, *z*; (iv) −*x*+2, −*y*+1, −*z*+1; (v) −*x*+1, −*y*+1, −*z*+1; (vi) −*x*+1, −*y*, −*z*; (vii) *x*, *y*, *z*+1; (viii) −*x*+2, −*y*, −*z*+1.

### Calcium hydrogen citrate trihydrate (CAHCIT_DFT) Crystal data

**Table:**

[Ca(C_6_H_6_O_7_)(H_2_O)_3_]	*c* = 23.8176 Å
*M**_r_* = 284.2	β = 116.7700°
Monoclinic, *P*2_1_/*c*	*V* = 1045.35 Å^3^
*a* = 8.7955 Å	*Z* = 4
*b* = 5.5891 Å	

### Calcium hydrogen citrate trihydrate (CAHCIT_DFT) Data collection

**Table:**

*h* = →	*l* = →
*k* = →	

### Calcium hydrogen citrate trihydrate (CAHCIT_DFT) Fractional atomic coordinates and isotropic or equivalent isotropic displacement parameters (Å^2^)

**Table:**

	*x*	*y*	*z*	*U*_iso_*/*U*_eq_	
C1	0.76524	0.05348	0.37245	0.01640*	
C2	0.95140	0.08798	0.41838	0.01460*	
C3	0.03847	0.27647	0.39546	0.01420*	
C4	0.04728	0.18511	0.33591	0.01370*	
C5	0.22520	0.32338	0.44588	0.01720*	
C6	0.23352	0.46010	0.50207	0.01570*	
O7	0.27344	0.46657	0.18178	0.02140*	
O8	0.65243	0.11625	0.38870	0.02060*	
O9	0.94176	0.49387	0.37832	0.01870*	
O10	0.09279	−0.02877	0.33519	0.01850*	
O11	0.01008	0.32908	0.29062	0.01980*	
O12	0.17785	0.12176	0.03732	0.02810*	
O13	0.69827	0.32564	0.49124	0.02930*	
O14	0.36036	0.36647	0.31711	0.02640*	
O15	0.58350	0.25887	0.10989	0.03520*	
O16	0.62980	0.41541	0.24756	0.02480*	
Ca17	0.14260	0.19608	0.22556	0.01080*	
H18	0.03696	0.85761	0.53607	0.02070*	
H19	−0.01870	0.08077	0.57649	0.02070*	
H20	0.10289	0.46976	0.59127	0.02070*	
H21	0.71042	0.84819	0.53720	0.02070*	
H22	0.70600	0.57636	0.57478	0.02070*	
H23	0.30870	0.75353	0.54836	0.03810*	
H24	0.53176	0.71932	0.66252	0.03330*	
H25	0.62196	0.47982	0.65997	0.03330*	
H26	0.50523	0.07373	0.72512	0.03330*	
H27	0.65711	0.24461	0.76515	0.03330*	
H28	0.30452	0.83594	0.37404	0.04570*	
H29	0.51280	0.12685	0.61841	0.04570*	

### Calcium hydrogen citrate trihydrate (CAHCIT_DFT) Bond lengths (Å)

**Table:**

C1—C2	1.517	O13—C6^iii^	1.317
C1—O7^i^	1.274	O13—H23^iii^	1.018
C1—O8	1.266	O14—H24^iii^	0.975
C2—C3^ii^	1.540	O14—H25^iii^	0.992
C2—H18^iii^	1.086	O15—H28^i^	0.981
C2—H19^iv^	1.091	O15—H29^viii^	0.974
C3—C2^v^	1.540	O16—H26^viii^	0.981
C3—C4	1.542	O16—H27^viii^	0.971
C3—C5	1.559	H18—C2^iii^	1.086
C3—O9^v^	1.433	H19—C2^iv^	1.091
C4—O10	1.263	H20—O9^iii^	0.988
C4—O11	1.265	H21—C5^iii^	1.094
C5—C6	1.514	H22—C5^iii^	1.091
C5—H21^iii^	1.094	H23—O13^iii^	1.018
C5—H22^iii^	1.091	H24—O14^iii^	0.975
C6—O12^vi^	1.235	H25—O14^iii^	0.992
C6—O13^iii^	1.317	H26—O16^vi^	0.981
O7—C1^vii^	1.274	H27—O16^vi^	0.971
O9—C3^ii^	1.433	H28—O15^vii^	0.981
O9—H20^iii^	0.988	H29—O15^vi^	0.974
O12—C6^viii^	1.235		

Symmetry codes: (i) −*x*+1, *y*−1/2, −*z*+1/2; (ii) *x*+1, *y*, *z*; (iii) −*x*+1, −*y*+1, −*z*+1; (iv) −*x*+1, −*y*, −*z*+1; (v) *x*−1, *y*, *z*; (vi) *x*, −*y*+1/2, *z*+1/2; (vii) −*x*+1, *y*+1/2, −*z*+1/2; (viii) *x*, −*y*+1/2, *z*−1/2.

### Calcium hydrogen citrate trihydrate (CAHCIT_DFT) Hydrogen-bond geometry (Å, º)

**Table:**

*D*—H···*A*	*D*—H	H···*A*	*D*···*A*	*D*—H···*A*
O15^vi^—H29···O8^iv^	0.974	1.940	2.891	164.6
O15^vii^—H28···O10^ix^	0.979	1.831	2.806	173.5
O16^vi^—H27···O7^iii^	0.971	1.970	2.926	167.6
O16^vi^—H26···O7^vi^	0.981	1.838	2.817	176.3
O14^iii^—H25···O15^vi^	0.992	1.719	2.702	170.9
O14^iii^—H24···O8^iii^	0.975	1.782	2.742	167.1
O13^iii^—H23···O8^iii^	1.018	1.560	2.571	171.4
O9^iii^—H20···O12^vi^	0.988	1.764	2.749	174.9

Symmetry codes: (iii) −*x*+1, −*y*+1, −*z*+1; (iv) −*x*+1, −*y*, −*z*+1; (vi) *x*, −*y*+1/2, *z*+1/2; (vii) −*x*+1, *y*+1/2, −*z*+1/2; (ix) *x*, *y*+1, *z*.
